# Lack of association between interleukin-1 receptor antagonist gene 86-bp VNTR polymorphism and ischemic stroke: A meta-analysis

**DOI:** 10.1097/MD.0000000000011750

**Published:** 2018-08-03

**Authors:** Yujiao Yang, Wenhui Wu, Long Wang, Yi Ding

**Affiliations:** a Department of Geriatrics; b Department of Hemodialysis, The Third Affiliated Hospital of Soochow University, Changzhou, Jiangsu, China.

**Keywords:** interleukin-1 receptor antagonist, ischemic stroke, meta-analysis, variable number tandem repeat

## Abstract

**Objective:**

The results of published studies which examined the association between variable number tandem repeat (VNTR) polymorphism of interleukin-1 receptor antagonist (IL-1RN) and ischemic stroke (IS) are conflicting. Thus, we performed a meta-analysis to examine the potential association between IL-1RN VNTR polymorphism and IS risk.

**Methods:**

A systematic literature search of PubMed, Embase, Medline, Web of Science, Cochrane Library, Chinese Biomedical Literature Database, Chinese National Knowledge Infrastructure, CQVIP, and WANFANG Database identified 10 studies with 2331 cases and 3335 controls. The pooled odds ratio (OR) with 95% confidence interval (95% CI) was calculated to evaluate the strength of the association. Subgroup analysis and meta-regression analysis were used to investigate the potential sources of heterogeneity. Begg funnel plots were used to explore the publication bias.

**Results:**

In this study, no enough proof was found to prove the association between IL-1RN 86-bp VNTR polymorphism and IS risk with random-effects model in the homozygous model (1/1 vs 2/2, OR = 0.97, 95% CI = 0.50–1.87, *P*_heterogeneity_ = .00), the heterozygous model (1/2 vs 2/2, OR = 0.64, 95% CI = 0.41–1.01, *P*_heterogeneity_ = .10), the dominant model (1/1 + 1/2 vs 2/2, OR = 0.85, 95% CI = 0.51–1.42, *P*_heterogeneity_ = .02), the recessive model (1/1 vs 1/2 + 2/2, OR = 0.69, 95% CI = 0.46–1.03, *P*_heterogeneity_ = .00), and allelic model (1 vs 2, OR = 1.24, 95% CI = 0.89–1.74, *P*_heterogeneity_ = .00). A marginally significant negative association was observed between IL-1RN 86-bp VNTR polymorphism and IS risk in the heterozygous model in the fixed-effects model (1/2 vs 2/2, OR = 0.71, 95% CI = 0.53–0.95, *P*_heterogeneity_ = .10). In subgroup analyses, similar association was found in the group whose control size was lower than 300.

**Conclusion:**

In conclusion, our results suggested that there was no sufficient evidence to support the association between IL-1RN 86-bp VNTR polymorphism and IS. Further large epidemiologic studies need to be done to confirm these findings.

## Abstract 2

Supplemental Digital Content is available in the text

## 1 Introduction

Stroke is one of the major causes of mortality and most common cause of disability worldwide.^[1,2]^ Approximately 83% of strokes are attributed to ischemic stroke (IS).^[3]^ Etiologically, IS is a multifactorial disease with the combination of several environmental and lifestyle factors. Previous study recommended that the genetic influence was an important tool for determining the risk of developing IS.^[4]^ Recently, a mounting evidence has shown that inflammatory cytokines play a crucial role in the development of IS.^[5,6]^ Weyrich et al reported that chronic low-grade inflammation and activation of the innate immune system are closely involved in the pathogenesis of IS.^[7]^ Inflammatory reactions have been identified in the pathogenesis of cerebral ischemia.^[8]^ So IS has been recognized as an inflammation-related disease.

Interleukin-1 (IL-1) is one of the proinflammatory cytokines produced by monocytes, macrophages and epithelial cells. The IL-1 family consists of the cytokines IL-1 alpha (IL-1α), IL-1 beta (IL-1β), and a specific receptor antagonist (IL-1RN).^[9]^ IL-1 gene complex, mapped on chromosome 2q13-14, including 3 linked genes which encode the secreted glycoproteins, IL-1α, IL-1β, and IL-1RN, respectively.^[10]^ IL-1α and IL-1β are proinflammatory cytokines, while IL-1RN, a 16 to 18 kD protein, is an important counter-inflammatory cytokine by competing with the binding of IL-1 to its receptor. There are a variable number of identical tandem repeats (VNTRs) in intron 2 of the IL-1RN, which contains 86 base pair nucleotide (rs2234663) sequence as repeating element. Five alleles, which are allele 1 (IL-1RN∗1) with 4 repeats, allele 2 (IL-1RN∗2) with 2 repeats, allele 3 (IL-1RN∗3) with 5 repeats, allele 4 (IL-1RN∗4) with 3 repeats, and allele 5 (IL-1RN∗5) with 6 repeats, has been identified. Tarlow et al showed that IL-1RN∗1 was the most common genotype, and then was the IL-1RN∗2. The remaining alleles (IL-1RN∗3, IL-1RN∗4, and IL-1RN∗5) occurred in <1% of the population.^[11]^ Accumulating genetic association studies have investigated the potential association of the IL-1RN VNTR with IS risk. However, the results of these studies remain controversial. The IL-1RN∗2 polymorphism has been recognized as a genetic risk factor for atherosclerosis and coronary artery disease, which are closely related with IS.^[12,13]^ Worrall et al confirmed that IL-1RN∗2 was associated with IS among Caucasians.^[14]^ However, Balding et al found no significant interaction between IL-1RN VNTR polymorphism frequencies and IS.^[15]^ Here we perform a meta-analysis based on 10 eligible pooled data to further validate the relationship between the IL-1RN VNTR polymorphism and IS susceptibility.

## 2 Materials and methods

### 2.1 Literature search

We systematically searched on the databases of PubMed, Embase, Medline, Web of Science, Cochrane Library, Chinese Biomedical Literature Database, Chinese National Knowledge Infrastructure (CNKI), CQVIP and WANFANG Database up to February 22, 2018, using the following medical subject headings (MeSH) or search terms: (“stroke” or “cerebrovascular disease” or “ischemic stroke” or “brain infarction”) and (“polymorphism∗” or “variation” or “VNTR” or “variable number tandem repeat”) and (“IL-1RN” or “IL-1Ra” or “interleukin 1 receptor antagonist”). The bibliographies of previous similar meta-analyses were also checked for additional relevant publications. We restricted our review to English and Chinese studies, owing to translation difficulties and lack of resources for review. Ethical approval or informed written consent was not applicable in our article because it was a meta-analysis of previously published studies.

### 2.2 Inclusion and exclusion criteria

The inclusion criteria for studies were as follows: independent case–control studies; studies that evaluated the association between IL-1RN 86-bp VNTR polymorphism and IS susceptibility; studies that provided complete data regarding genotype and allele frequencies. The exclusion criteria were as follows: insufficient data regarding genotypes and allele frequencies; the genotype distribution of the control population did not conform to Hardy–Weinberg equilibrium (HWE), and overlapping publications.

### 2.3 Data extraction

Three researchers (YY, WW, and LW) extracted information independently including the first author's name, year of publication, country, source of controls, genotype number and allele number in cases and controls, and HWE in controls. Disagreement was resolved through team discussion.

### 2.4 Quality assessment

Two researchers (YY and WW) performed independent study quality assessment using the 9-point Newcastle–Ottawa scale (NOS).^[16]^ The quality of each study was assessed based on appropriateness of selection, comparability, and exposure. NOS scores ranged from 0 to 9, and a score of 6 or greater indicate high quality.

### 2.5 Statistical analysis

The association between IL-1RN 86-bp VNTR polymorphism and IS was compared using the odds ratio (OR) corresponding to a 95% confidence interval (95% CI). Five different comparison models were used as follows: the 1/1 versus the 2/2 genotypes (homozygous model), the 1/1 + 1/2 versus the 2/2 genotypes (dominant model), the 1/2 versus the 2/2 genotypes (heterozygous model), the 1/1 versus the 2/2 + 1/2 genotypes (recessive model), the 1 versus 2 alleles (allele model).

The HWE was tested by Pearson goodness-of-fit Chi-squared test. Two studies were excluded due to not in HWE. Heterogeneity between studies was assessed using *P*-values for the *Q* statistic, *H* statistic, and *I*^*2*^ values. The heterogeneity was considered significant when *P* < .05, or H > 1.5, or *I*^*2*^ > 50%. *I*^*2*^ was calculated to describe the percentage of variation caused by the heterogeneity: 0% to 25%, no heterogeneity; 25% to 50%, moderate heterogeneity; 50% to 75%, large heterogeneity; and 75% to 100%, extreme heterogeneity.^[17–19]^ The selection of a statistical model was based on the question of which model fits the distribution of effect sizes, and takes account of the relevant sources of error. This meta-analysis was performed using a random-effects model, which provided more conservative estimated effects.^[20]^ We also used subgroup analysis based on the region, source of control, control size, and case size. And meta-regression was used to explore reasons for heterogeneity. Sensitivity analyses were performed to assess the stability of the results. We also performed a cumulative meta-analysis to detect the result trends. To test for publication bias, Begg funnel plot and Egger linear regression test were applied.^[21]^ All statistical tests for this meta-analysis were performed with STATA 14.0 software (Version MP 14.0; Stata Corporation, College Station, TX).

## 3 Results

### 3.1 Eligible studies and study characteristics

**Figure 1:**
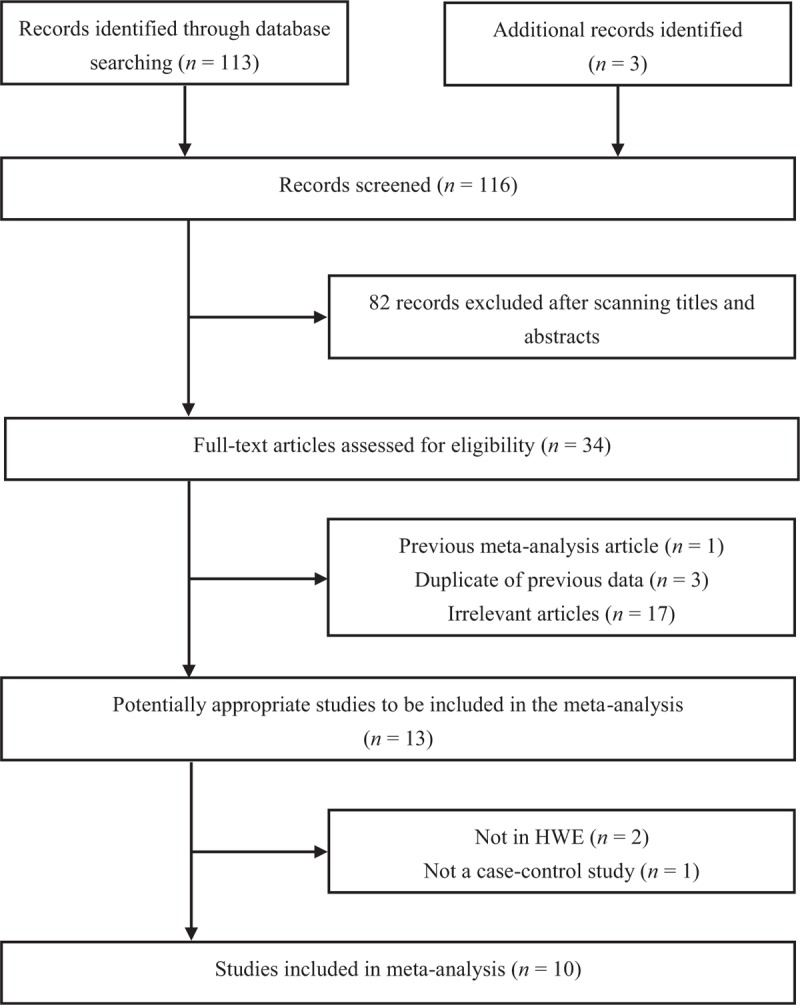
Flow diagram of study identification.

Our literature search initially identified 116 articles by using the prespecified search strategy. Figure 1 showed the process of retrieving eligible studies. After title and abstract evaluation, we were left with 34 selected articles to be assessed by viewing full-text in the end. Three of these were eliminated due to reporting duplicate records, 1 was excluded because it was a meta-analysis and the others were excluded for their irrelevance to our study. Of the remaining 13 publications,^[14,18,19,22–31]^ 2 were eliminated for the unequilibrium of HWE in the control group,^[18,19]^ and 1 was eliminated for not a case–control study.^[29]^ As a result, 10 articles met eligibility criteria for this analysis, which included 3335 controls and 2331 IS patients. One publication included 2 separate case–control studies was considered to be a study when we do the analysis.^[14]^ In term of study design, 7 studies had population-based source of controls,^[14,22,23,26–28,31]^ and 3 had both population-based and hospital-based source of controls.^[24,25,30]^ Five studies were designed among Asians,^[23,26,27,30,31]^ and 5 studies non-Asians.^[14,22,24,25,28]^ Two articles were in Chinese^[30,31]^ and other 8 were all in English (Table 1).^[14,22–28]^

**Table 1:**
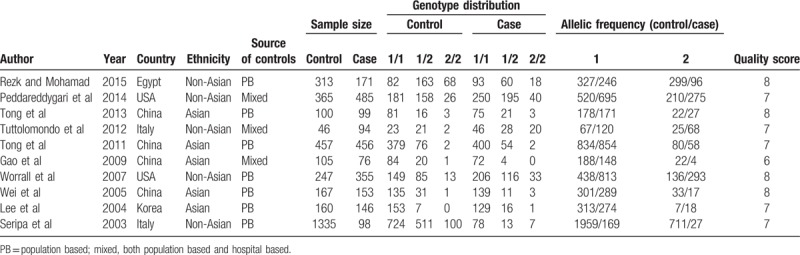
Studies and data included in this meta-analysis.

### 3.2 Meta-analysis results

**Figure 2:**
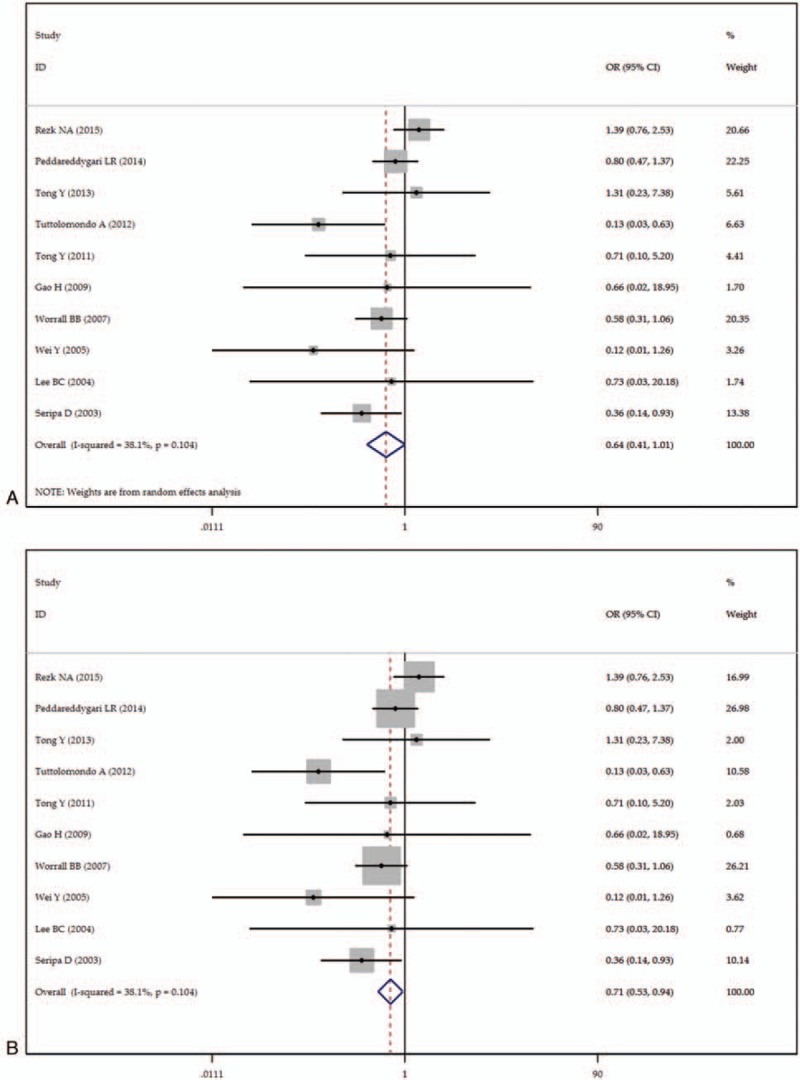
Forest plot of ischemic stroke risk associated with the interleukin-1 receptor antagonist gene 86-bp variable number tandem-repeat polymorphism (heterozygous comparison). (A) Results in random-effects model. (B) Results in fixed-effects model.

To evaluate the association between IL-1RN 86-bp VNTR polymorphism and IS risk, we performed both the overall meta-analysis and subgroup meta-analysis based on ethnicity, source of control, control size, and case size. Table 2 showed the main results of the meta-analysis. There are no significant associations in the homozygous model (1/1 vs 2/2, OR = 0.97, 95% CI = 0.50–1.87, *P*_heterogeneity_ = .00), the heterozygous model (1/2 vs 2/2, OR = 0.64, 95% CI = 0.41–1.01, *P*_heterogeneity_ = .10), the dominant model (1/1 + 1/2 vs 2/2, OR = 0.85, 95% CI = 0.51–1.42, *P*_heterogeneity_ = .02), the recessive model (1/1 vs 1/2 + 2/2, OR = 0.69, 95% CI = 0.46–1.03, *P*_heterogeneity_ = .00), and allelic model (1 vs 2, OR = 1.24, 95% CI = 0.89–1.74, *P*_heterogeneity_ = .00). There existed minor heterogeneity among studies in the heterozygous model (*I*^*2*^ = 38.1%). We also calculated the ORs and 95% CIs with fixed-effects model in the heterozygous model in our analysis for comparison. A marginally significant negative association was observed between IL-1RN 86-bp VNTR polymorphism and IS risk in the heterozygous model in the fixed-effects model (1/2 vs 2/2, OR = 0.71, 95% CI = 0.53–0.95, *P*_heterogeneity_ = .10) (Fig. 2).

**Table 2:**
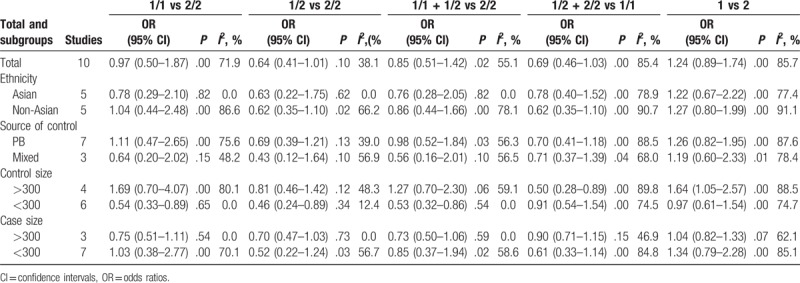
Pooled ORs and 95% CIs of the association between interleukin-1 receptor antagonist variable number tandem repeat polymorphism and ischemic stroke.

We further performed subgroup analysis according to the region, source of control, control size, and case size. We still found no association between IL-1RN 86-bp VNTR polymorphism and IS risk in non-Asians, Asians, in population-based control group, mixed control group, and in groups whose case size was lower or higher than 300. However, in the stratified meta-analysis based on control size, significant association between IL-1RN 86-bp VNTR polymorphism and IS susceptibility was observed in the group whose sample size was higher than 300 in the recessive model (1/1 vs 1/2 + 2/2, OR = 0.50, 95% CI = 0.28–0.89, *P*_heterogeneity_ = .00), and the allelic model (1 vs 2, OR = 1.64, 95% CI = 1.05–2.57, *P*_heterogeneity_ = .00). However, in the subgroup whose sample size was lower than 300, we observed a decreased risk in the homozygous model (OR = 0.54, 95% CI = 0.33–0.89, *P*_heterogeneity_ = .65), heterozygous model (OR = 0.46, 95% CI = 0.24–0.89, *P*_heterogeneity_ = .34), and the dominant model (OR = 0.53, 95% CI = 0.32–0.86, *P*_heterogeneity_ = .54), but no association was observed in the recessive model (OR = 0.91, 95% CI = 0.54–1.54, *P*_heterogeneity_ = .00), or the allelic model (OR = 0.97, 95% CI = 0.61–1.54, *P*_heterogeneity_ = .00).

### 3.3 Heterogeneity evaluation

A minor heterogeneity has been observed among studies in the heterozygote model (OR = 0.64, 95% CI = 0.41–1.01, *P*_heterogeneity_ = .10, *I*^*2*^ = 38.1%). However, significant heterogeneity was found in the other four genetic models (1/1 vs 2/2, *P*_heterogeneity_ = .00, *I*^*2*^ = 71.9%; 1/2 + 1/1 vs 2/2, *P*_heterogeneity_ = .02, *I*^*2*^ = 55.1%; 1/2 + 2/2 vs 1/1, *P*_heterogeneity_ = .00, *I*^*2*^ = 85.4%; 1 vs 2, *P*_heterogeneity_ = .00, *I*^*2*^ = 85.7%). The results of the stratified analysis by ethnicity, source of controls, control size, and case size revealed that non-Asian group, population-based control group, and group whose case size lower than 300 were contributed to the heterogeneity. We also conducted meta-regression to assess the extent to which variables explained the heterogeneity. However, the results revealed that the heterogeneity could not be explained by publication year, ethnicity of the population, source of controls, control size, case size, and total size.

### 3.4 Sensitivity analysis and cumulative meta-analysis

**Figure 3:**
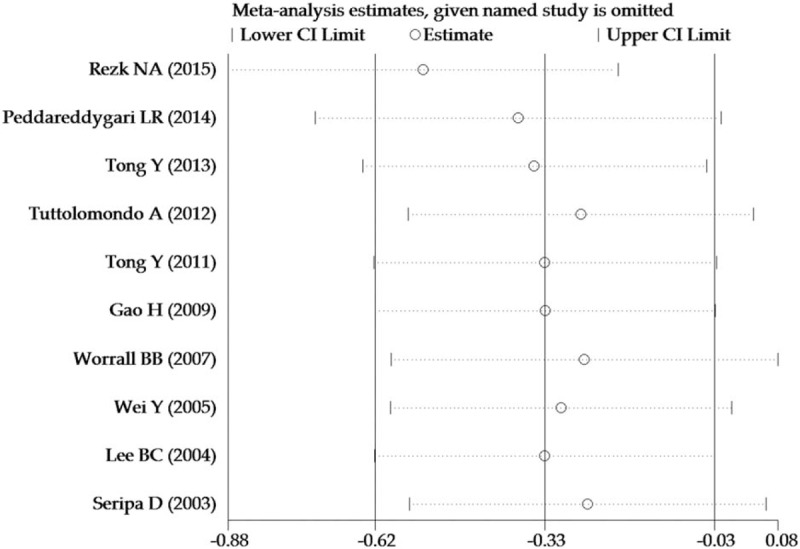
Sensitivity analysis of the summary OR coefficients on the association between interleukin-1 receptor antagonist 86-bp variable number tandem-repeat polymorphism and ischemic stroke risk.

**Figure 4:**
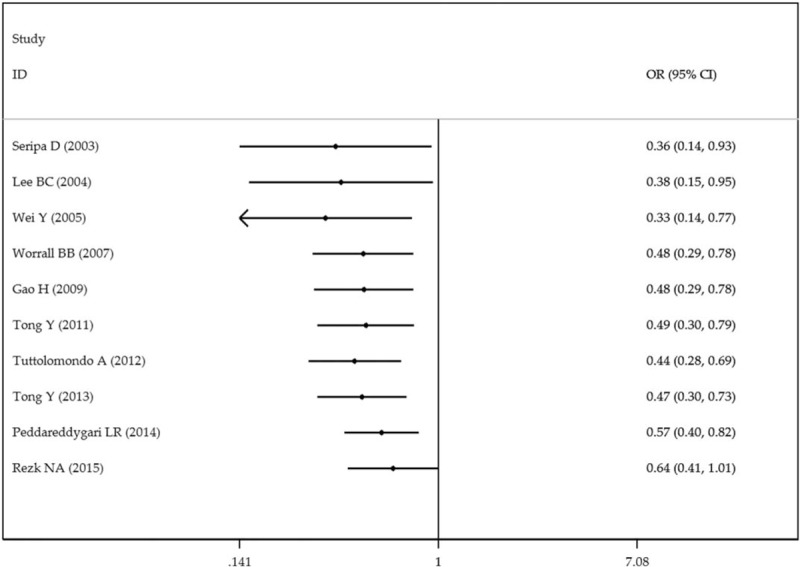
The heterozygote model in cumulative meta-analysis by publication year.

Leave-one-out sensitivity analysis was performed to measure the effects of individual research on the meta-analysis results by omitting one study at a time. Then we recalculated the ORs and 95% CIs. Sensitivity analysis revealed that the study of Rezk and Mohamad was the main cause of heterogeneity^[22]^ (Fig. 3). When the study was omitted, the heterogeneity had significantly fallen and a negative association between IL-1RN 86-bp VNTR polymorphism and IS risk was found under both the heterozygous model (1/2 vs 2/2, OR = 0.57, 95% CI = 0.40–0.82, *P*_heterogeneity_ = .39, *I*^*2*^ = 5.2%) and the dominant model (1/1 + 1/2 vs 2/2, OR = 0.72, 95%CI = 0.52–0.99, *P*_heterogeneity_ = .53, *I*^*2*^ = 0.0%). The cumulative meta-analysis was performed by accumulating the studies sorted by publication year. Under the heterozygote model, the cumulative meta-analysis displays that with the included study accumulated, the OR value showed corresponding increases (Fig. 4). But this trend was not observed in the other 4 models.

### 3.5 Publication bias test results

**Figure 5:**
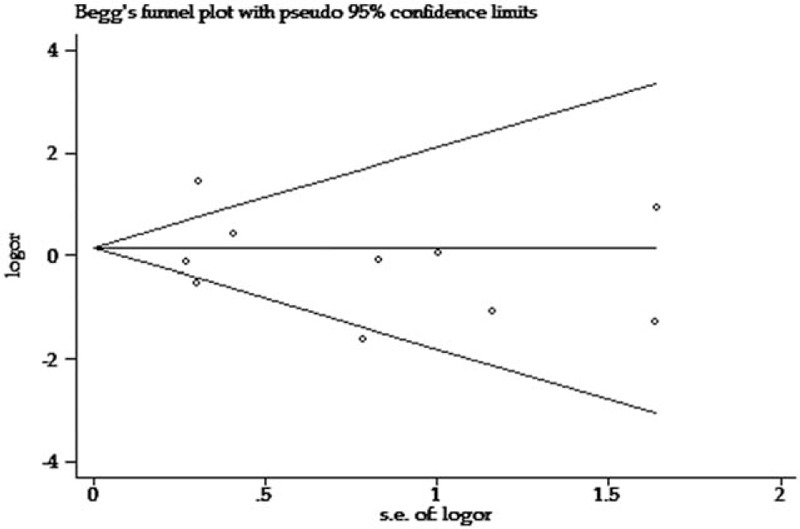
Begg funnel plot for assessment of publication bias for interleukin-1 receptor antagonist 86-bp variable number tandem-repeat polymorphism.

Begg funnel plot and Egger test were performed to assess the publication bias in the included studies. The results did not show any evidence of publication bias (Fig. 5) (Begg test: *P* = .54 for dominant model, *P* = .47 for heterozygous model, *P* = 1.00 for dominant model, *P* = .86 for recessive model, *P* = .86 for allelic model, respectively; Egger test: *P* = .49 for dominant model, *P* = .25 for heterozygous model, *P* = .40 for dominant model, *P* = .67 for recessive model, *P* = .82 for allelic model, respectively).

## 4 Discussion

The present meta-analysis suggested that no sufficient evidence was found to support the correlation between IL-1RN VNTR polymorphism and IS risk. However, a significantly decreased risks of IS between the IL-1RN 1/2 group and the 2/2 group were observed in the fixed-effects model. After we omitted the study of Rezk and Mohamad, the heterogeneity fell significantly and a similar outcome of statistic association between the IL-1RN 1/2 group and the 2/2 group was found. In addition, the results of our stratified analysis indicated that IL-1RN VNTR polymorphism was associated with decreased risks of IS in homozygous model, heterozygous model, and dominant model in groups whose control size was <300. However, due to the limitation in our study, these results should be interpreted with caution.

Stroke ranks as the second leading cause of death and is reported to be the major cause of permanent disability worldwide. Approximately 87% of stroke patients suffer from IS, which is a complex multifactorial disease with genetic components, environmental triggers, and gene–environment interactions involved in its etiology.^[32,33]^ IL-1 is an important proinflammatory cytokine contribute to the pathophysiology of IS. IL-1Ra, which is a naturally competitive inhibitor of IL-1, inhibits the activity of IL-1 by binding to receptor IL-1R1 and preventing its association for signaling. Several studies have suggested a neuro-protection role of IL-1Ra in IS. In brain ischemia models, peripherally administered rhIL-1ra inhibits brain damage^[34]^ and overexpression of IL-1RN reduces ischemic brain injury.^[35]^ In 2006, Emsley et al reported that compared to the placebo treated acute stroke patients, clinical outcomes at 3 months in the intravenous recombinant human IL-1Ra-treated group were better.^[36]^ The IL-1Ra gene (IL-1RN) contains an 86-bp VNTR polymorphism in intron 2. Homozygotes for allele 2 were found to have more prolonged and more intense inflammatory responses than other genotypes.^[37]^ It is reported that patients with IL-1RN allele 2 had slightly but significantly reduced mucosal IL-1Ra concentrations.^[38]^ Decreased production of total IL-1Ra protein in cultured PBMCs from both ulcerative colitis patients and controls were also observed.^[39]^ And the IL-1RN allele 2 is also reported to be associated with higher circulating IL-1β levels.^[40]^ All of these resulted in a lower IL-1Ra/IL-1β ratio and was associated with the initiation of a proinflammatory response. The IL-1RN VNTR polymorphism has been reported to be correlated with increased risks to develop IS. However, the published results regarding the association are conflicting. The better understanding of the association between IL-1RN VNTR polymorphism and IS risk can provide novel insights into the prevention and early diagnosis of IS. So we attempted to summarize the current evidence on the association between IL-1RN VNTR polymorphism and IS risk. And no significant difference was found among 5 genetic models in the random-effect model in our meta-analysis.

However, we observed a higher IS risk in the IL-1RN 2/2 group than the 1/2 group in the fixed-effects model. And the conclusions remained same when we omitted the study of Rezk and Mohamad which accounted for the heterogeneity according to the sensitivity analysis. The similar results could also be identified in subgroups whose control size was <300. These results suggested that allele 2 of IL-1RN may be involved in the susceptibility to stroke. And this effect could be explained by the reduced expression of the IL-1Ra molecule in individuals. However, the fixed-effects model starts with the assumption that the true effect size is the same in all studies, which is implausible in many systematic reviews. And according to the rule of meta-analysis, it makes sense to use the fixed-effects model, if 2 conditions are met. First, it is believed that all the studies included in the analysis are functionally identical. Second, the study goal is to compute the common effect size for the identified population, and not to generalize to other population. Multiple testing could increase the probability of the overall likelihood of a type I error, which is an inevitably existed problem in meta-analysis.^[20]^ The significant results found in the heterozygous model in our study was minimal. And this marginally significant association could be a false-positive result due to multiple testing, rather than reflecting a true association. The results of the decreased risks of IS between the IL-1RN 1/2 group and the 2/2 group of fixed-effects model in this meta-analysis should be interpreted with caution. The study of Rezk and Mohamad was conducted to investigate the influence of IL-1 cluster gene polymorphisms on the susceptibility of acute stroke and its outcomes in Egyptian patient.^[22]^ The control group was ethnic origin, age, and sex matched with the stroke group. However, we only extracted information of the cases of IS in our study, which would lead to potential imbalances between groups. This might explain part of the result in sensitivity analysis. We also observed minor heterogeneity and decreased risks of IS between the IL-1RN 1/2 group and the 2/2 group in subgroups whose control size was <300. Interestingly, the study of Rezk and Mohamad was included in the subgroups whose control size was >300. And we assumed that the similar results in subgroups whose control size was <300 might be attributed to heterogeneity caused by the study of Rezk and Mohamad.

A meta-analysis by Zou et al (2015), in which 12 studies with 2814 cases and 2986 controls were included, declared no associations of IL-1RN polymorphisms on stroke risk.^[41]^ However, stroke has a number of different subtypes. Both IS and hemorrhagic stroke were included in the previous meta-analysis. And they did not perform the subgroup analysis based on stroke type. There was a significant difference among the pathogenesis, clinical feature, and prognosis between IS and hemorrhagic stroke. The hemorrhagic stroke involves the rupturing of a vessel, causing the leakage of blood into the parenchyma. Conversely, the basic mechanism of IS is the cessation or diminution of flow to regions of the brain.^[42,43]^ So it may not be appropriate to generalize the results on stroke into IS. As the meta-analysis conducted in 2015 covered various stroke types, the association of IL-1RN VNTR polymorphism and IS risk was still not clarified. Therefore, we carried out this meta-analysis to further investigate the role of IL-1RN polymorphisms on IS. Furthermore, our meta-analysis has larger number of participants (10 studies with 3335 controls and 2331 IS patients) than the previous one due to the inclusion of more databases in the search strategy. The substantially large sample size ensures the reliability of the results.

When interpreting the results, attention should be paid to some limitations in the current meta-analysis. Firstly, heterogeneity was detected in all the 5 genetic models, suggesting that results should be interpreted with caution. Secondly, only case–control studies were included in the study, and the sample sizes and the number of studies in this meta-analysis are limited. More credible evidences are required to detect the association between IL-1RN VNTR polymorphism and IS. Thirdly, we only enrolled studies related to the alleles containing 4 and 2 repeats. The other possible 3 alleles (allele 3/4/5) at the IL-1Ra locus were not included in this study.

In conclusion, our results suggested that the IL-1RN VNTR polymorphism was not associated with IS risk. However, allele 2 of IL-1RN may be involved in the susceptibility to stroke. The relationship between IL-1RN VNTR polymorphism and IS risk must be evaluated in future studies.

## 5 Conclusion

The results of the current meta-analysis suggested that IL-1RN 86-bp VNTR polymorphism is not related to IS. Further studies with larger sample sizes should be performed to confirm these findings.

## Author contributions

**Investigation:** Yujiao Yang, Wenhui Wu.

**Methodology:** Yujiao Yang, Wenhui Wu, Long Wang.

**Resources:** Yujiao Yang, Wenhui Wu.

**Writing – original draft:** Yujiao Yang, Wenhui Wu.

**Writing – review & editing:** Yi Ding.

## Supplementary Material

Supplemental Digital Content
