# Supplementary material for: Lack of association between interleukin-1 receptor antagonist gene 86-bp VNTR polymorphism and ischemic stroke: A meta-analysis
Source: Medicine (Baltimore). 2018 Aug 3;97(31):e11750. doi: 10.1097/MD.0000000000011750 (PMC6081086; doi:10.1097/MD.0000000000011750)
Supplement: Supplemental Digital Content [file medi-97-e11750-s001.docx]

**Supplementary Table 1.** Quality assessment of studies

| Studies | Selection  (score) | | | | Comparability  (score) | Exposure  (score) | | | Total  score |
| --- | --- | --- | --- | --- | --- | --- | --- | --- | --- |
|  | Adequate  definition of patient case | Representativeness of patients case | Selection of controls | Definition of control | Control for important factor or additional factor | Ascertainment of exposure (blinding) | Same method of ascertainment for participants | Non- response rate |  |
| Rezk et al.  (2015) | 1 | 1 | 1 | 1 | 2 | 1 | 1 | 0 | 8 |
| Peddareddygari et al.  (2014) | 1 | 1 | 0 | 1 | 1 | 1 | 1 | 0 | 6 |
| Tong et al.  (2013) | 1 | 1 | 1 | 1 | 2 | 1 | 1 | 0 | 8 |
| Tuttolomondo et al.  (2012) | 1 | 1 | 0 | 1 | 1 | 1 | 1 | 0 | 6 |
| Tong et al.  (2011) | 1 | 1 | 1 | 1 | 1 | 1 | 1 | 0 | 7 |
| Gao et al.  (2009) | 1 | 1 | 0 | 0 | 2 | 1 | 1 | 0 | 6 |
| Worrall et al.  (2007) | 1 | 1 | 1 | 1 | 2 | 1 | 1 | 0 | 8 |
| Wei et al.  (2005) | 1 | 1 | 1 | 1 | 2 | 1 | 1 | 0 | 8 |
| Lee et al.  (2004) | 1 | 1 | 1 | 1 | 1 | 1 | 1 | 0 | 7 |
| Seripa et al.  (2003) | 1 | 1 | 1 | 1 | 1 | 1 | 1 | 0 | 7 |

We assessed the methodological quality of included studies based on Newcastle-Ottawa Scale (NOS) for quality of case-control studies. A star system of the NOS (range, 0–9 stars) has been developed for the evaluation. Out of a maximum 9 stars, two studies had a quality score of 6, three studies had a star of 7, and three study had a quality star of 8.
